# Supplementary material for: Chemically activating MoS2 via spontaneous atomic palladium interfacial doping towards efficient hydrogen evolution
Source: Nat Commun. 2018 May 29;9:2120. doi: 10.1038/s41467-018-04501-4 (PMC5974284; doi:10.1038/s41467-018-04501-4)
Supplement: Supplementary file 1 — Supplementary Information [file 41467_2018_4501_MOESM1_ESM.pdf]

Supplementary Information for

Chemically activating MoS_2 via spontaneous atomic palladium interfacial doping towards efficient hydrogen evolution

Luo *et al.*

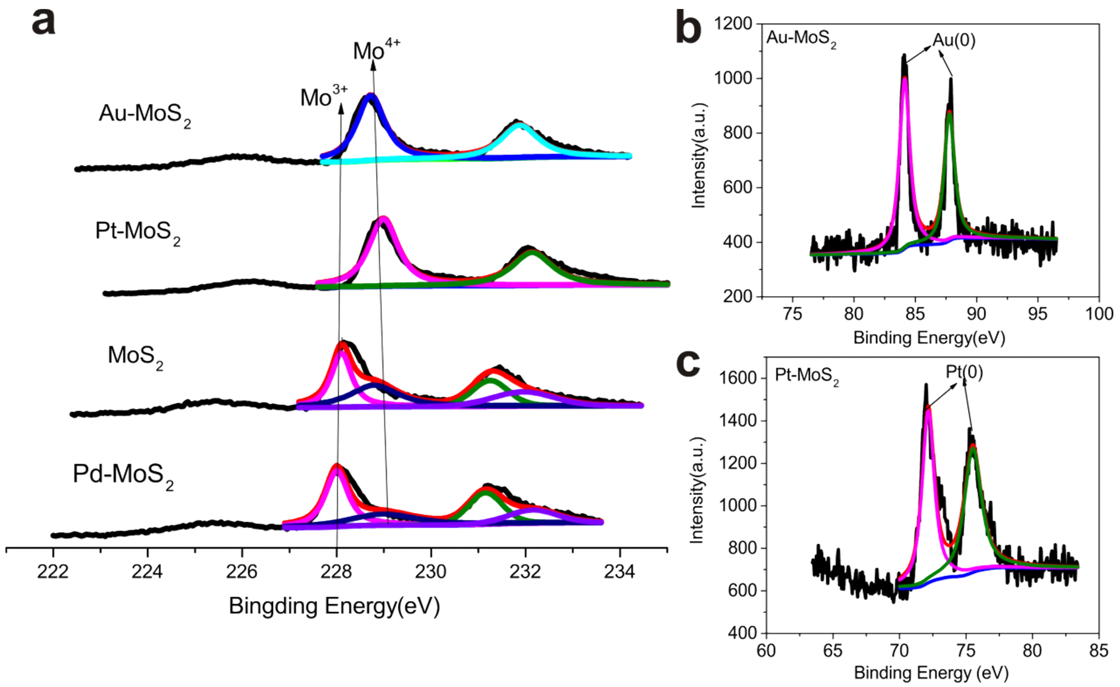

Supplementary Figure 1| XPS spectra for Au-MoS$_2$, Pt-MoS$_2$, MoS$_2$ and Pd-MoS$_2$. **a** High-resolution XPS results (Mo *3d* region) of Au-MoS$_2$, Pt-MoS$_2$, MoS$_2$ and Pd-MoS$_2$. **b** High-resolution XPS results (Au *3d* region) of Au-MoS$_2$. **c** High-resolution XPS results (Pt *3d* region) of Pt-MoS$_2$.

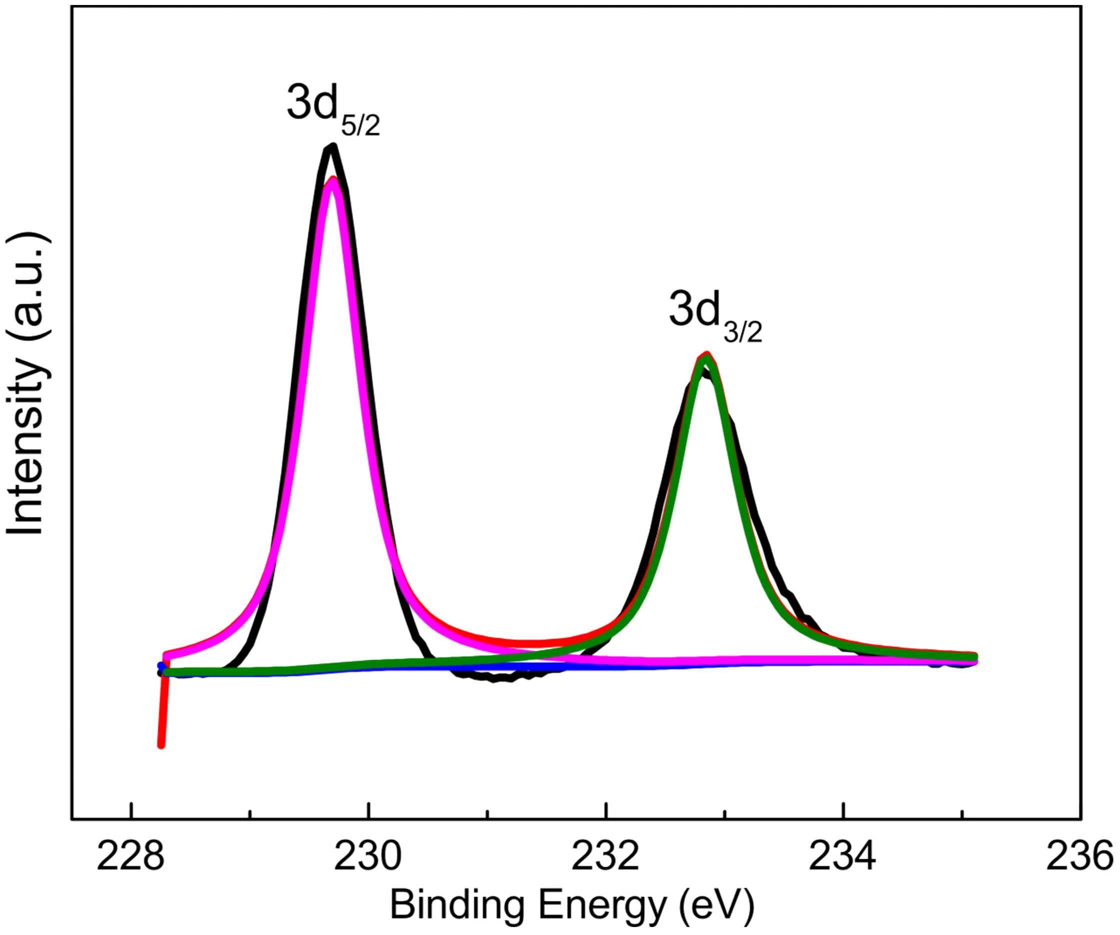

Supplementary Figure 2| XPS spectra for standard MoS_2. High-resolution XPS results (Mo *3d* region) of the standard MoS_2.

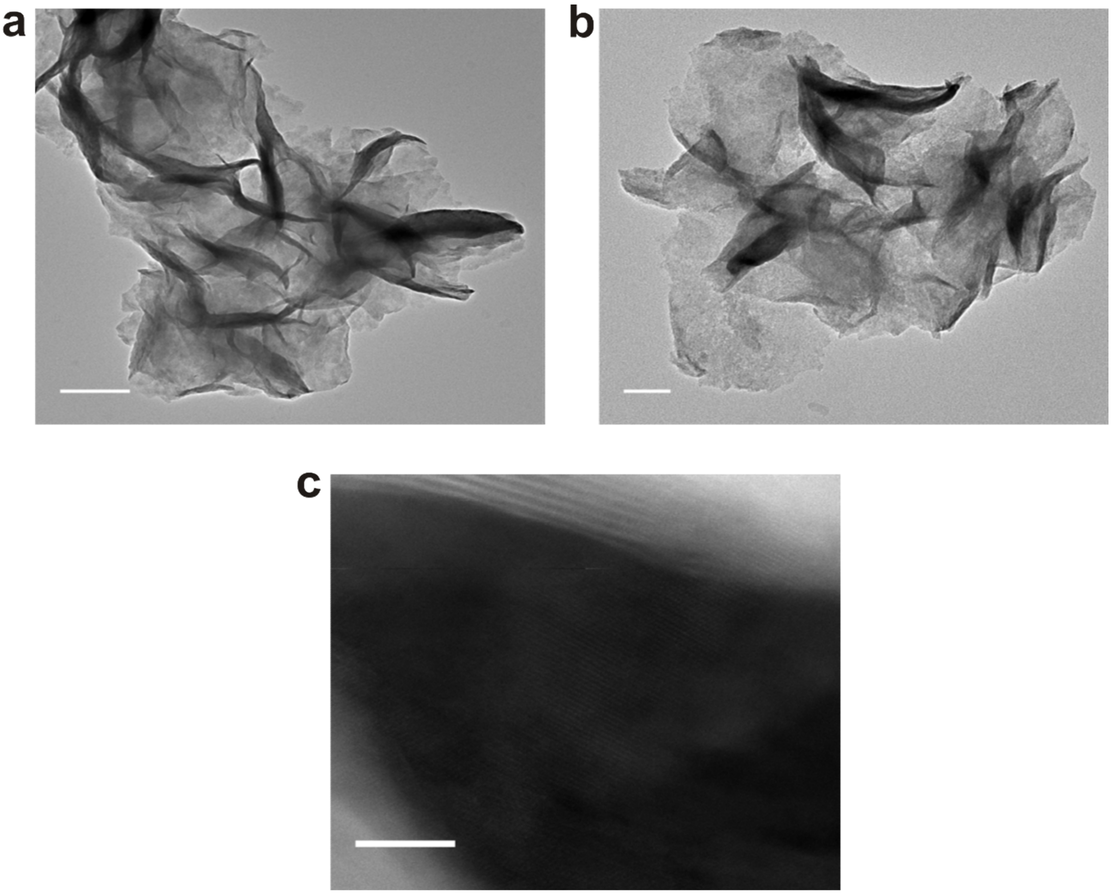

Supplementary Figure 3| TEM patterns. a TEM of MoS_2, the corresponding scale bar is 100 nm. **b**. TEM of Pd-MoS_2, the corresponding scale bar is 50 nm. **c**. TEM of Pd-MoS_2, the corresponding scale bar is 5 nm.

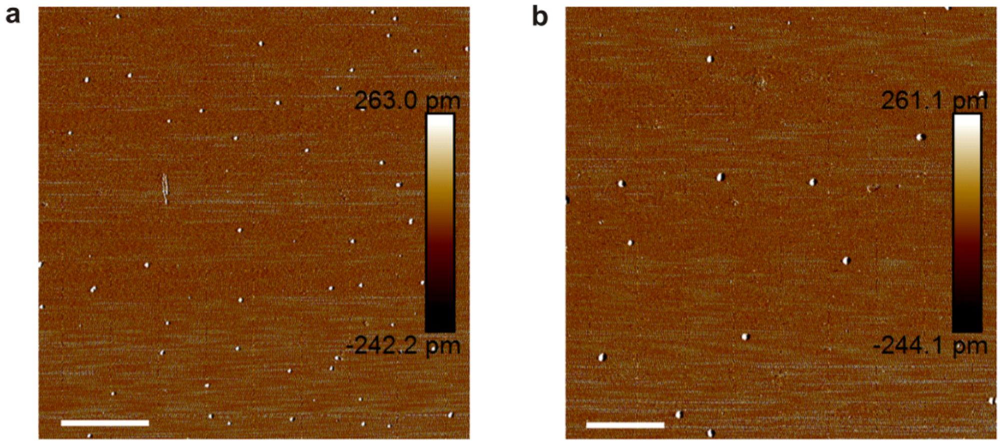

Supplementary Figure 4| Atomic force microscopy (AFM) patterns. a AFM of MoS_2, the corresponding scale bar is 2 um. **b** AFM of Pd-MoS_2, the corresponding scale bar is 1 um.

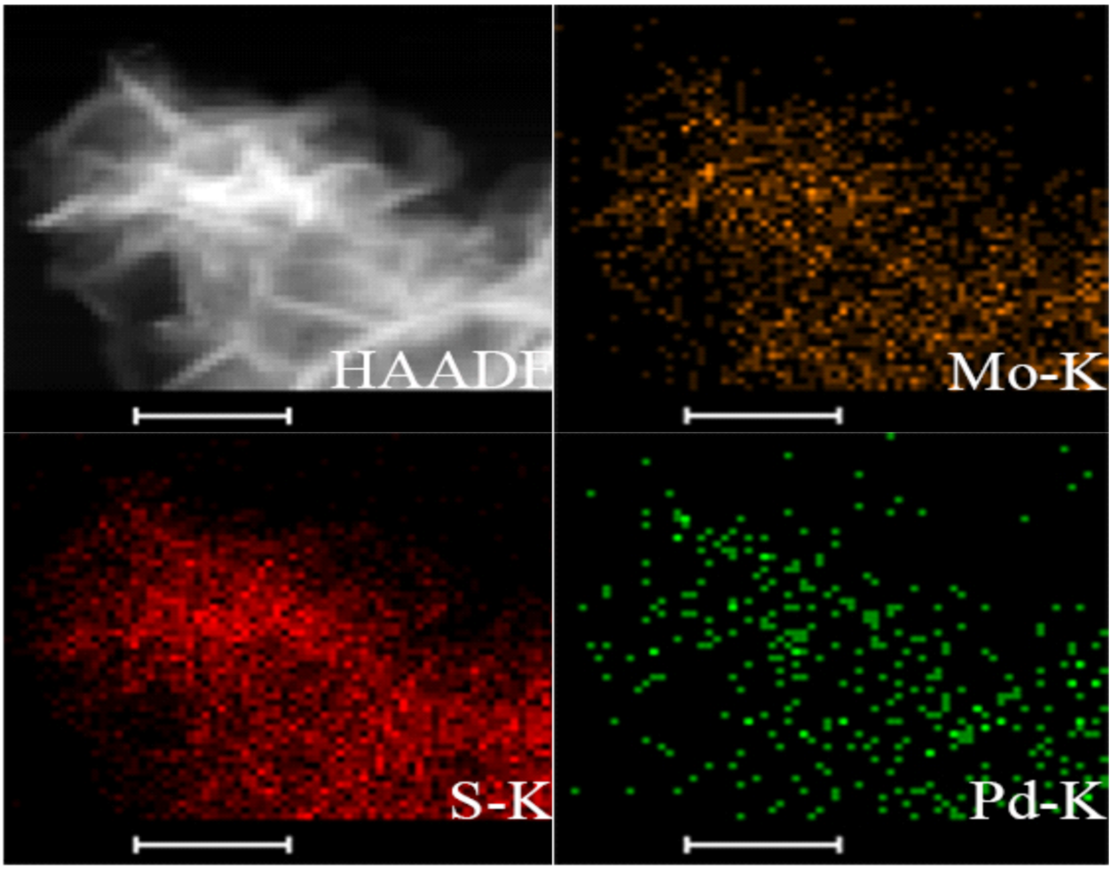

Supplementary Figure 5| HAADF-STEM patterns for Pd-MoS_2. HAADF and elemental mappings for S, Mo and Pd; the corresponding scale bars are 100 nm.

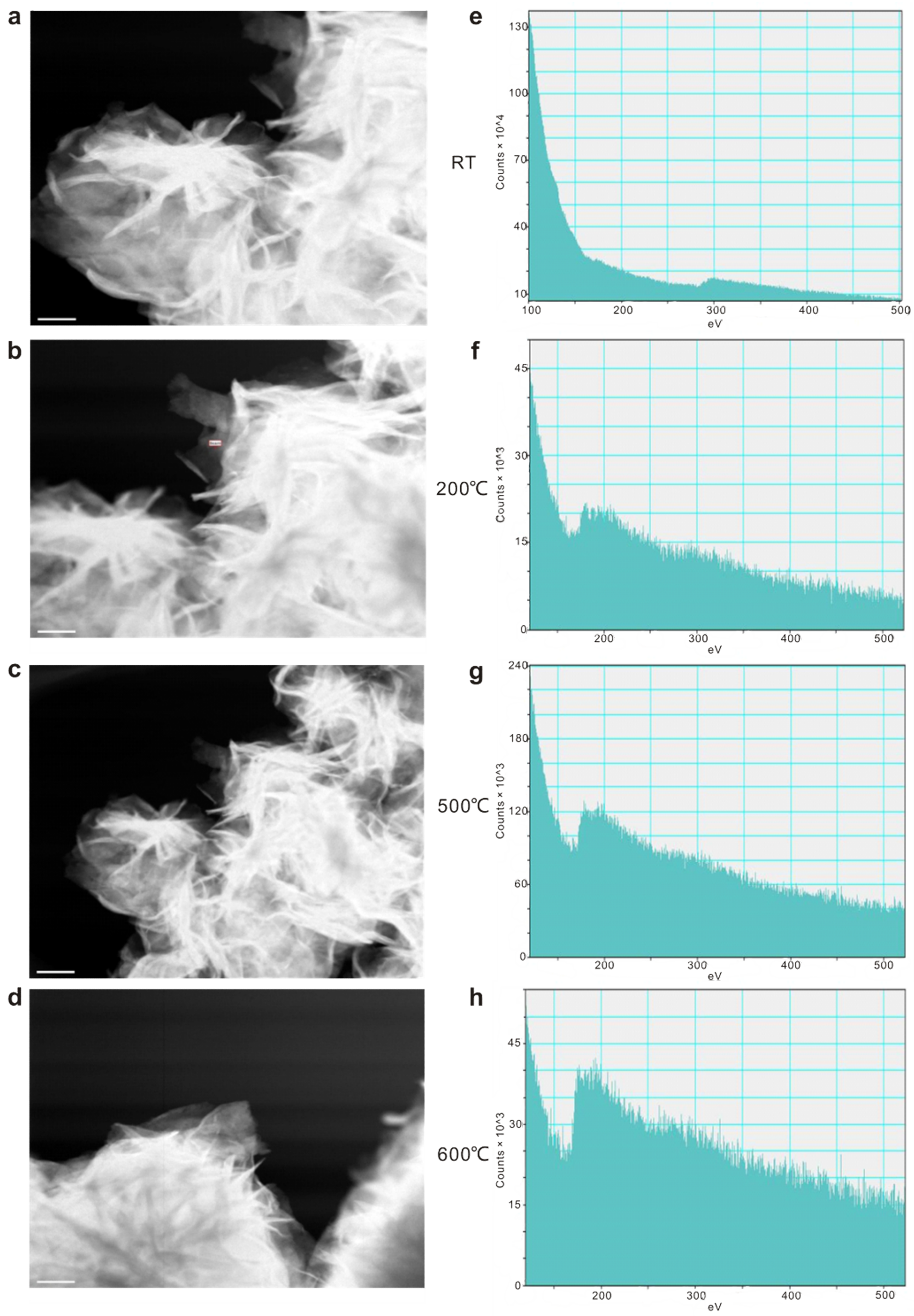

Supplementary Figure 6| In-situ heat treatment TEM coupling with EELS

In-situ heat treatment TEM coupling with EELS. a-d TEM patterns of Pd-MoS_2 taken at different temperatures (RT, 200℃, 500℃, 600℃), the corresponding scale bars are 100 nm. **e-h** EELS patterns of Pd-MoS_2 taken at different temperatures (RT, 200℃, 500℃, 600℃).

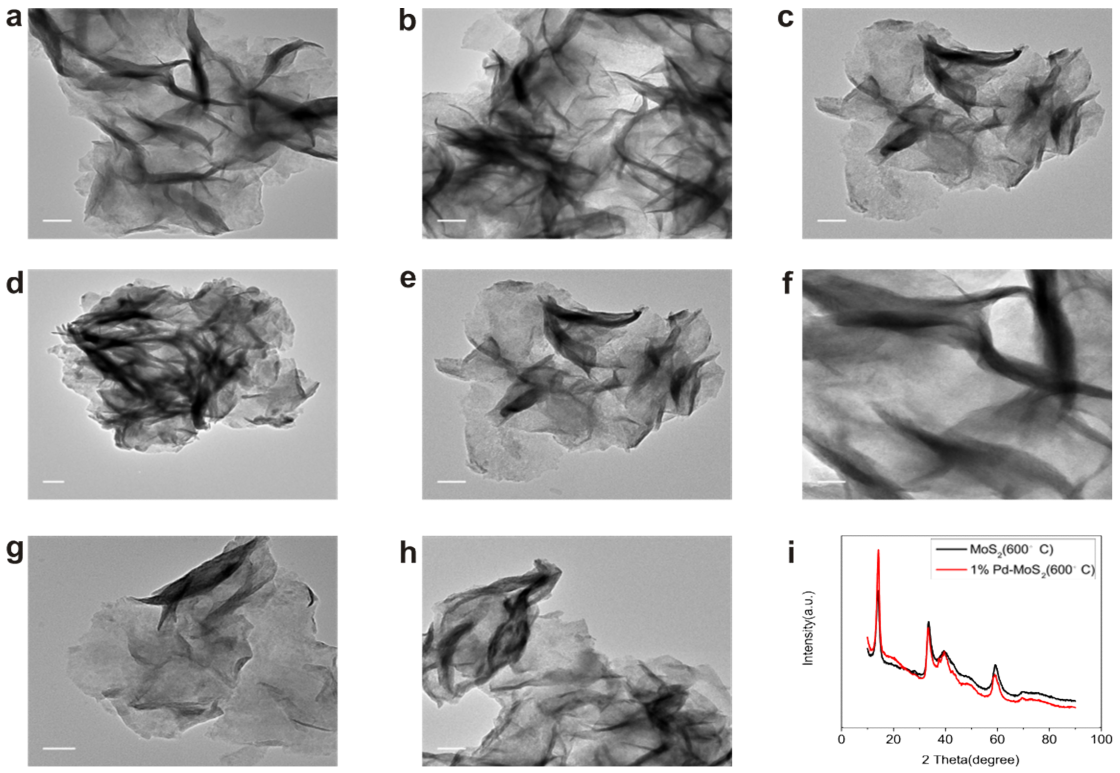

Supplementary Figure 7| TEM patterns and XRD pattern for samples with heat-treatment. a-c TEM images of 5%Pd-MoS_2 with 400 ℃ high-temperature calcinations, the scale bars are 50 nm. **d-f** TEM images of 5%Pd-MoS_2 with 600 ℃ high-temperature calcinations, the corresponding scale bars are 100, 50, and 20 nm, respectively. **g-h** TEM images of MoS_2 with 600 ℃ high-temperature calcinations, the scale bars are 50 nm. **i** XRD patterns of MoS_2 and 5%Pd-MoS_2 with 600 ℃ high-temperature calcination.

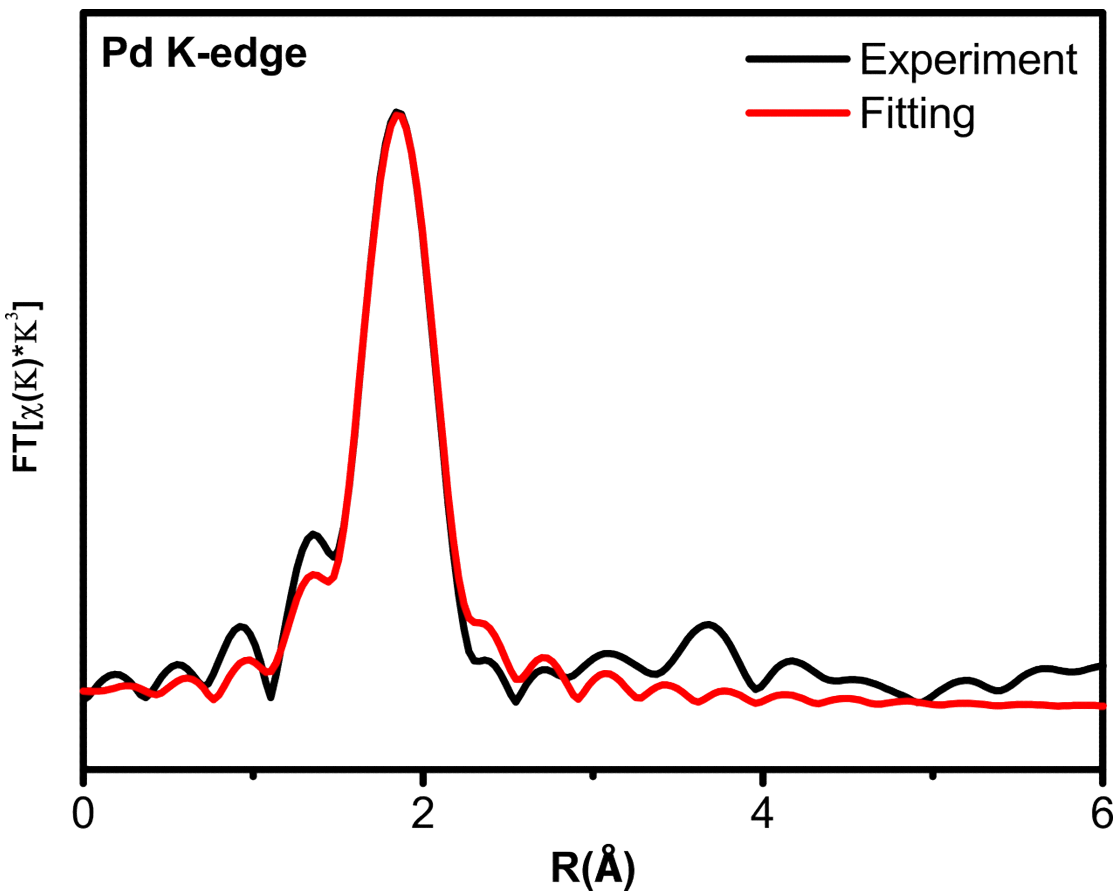

Supplementary Figure 8| EXAFS spectra. Fourier transforms of k^3-weighted Pd K-edge of EXAFS spectra of 1%Pd-MoS_2. The black solid line are experimental results, and the red dotted line are best-fit curves for R = 1.1–2.5 Å, using corresponding k^3 $\chi(k)$ functions in k = 3.0–12.0 $Å^{-1}$.

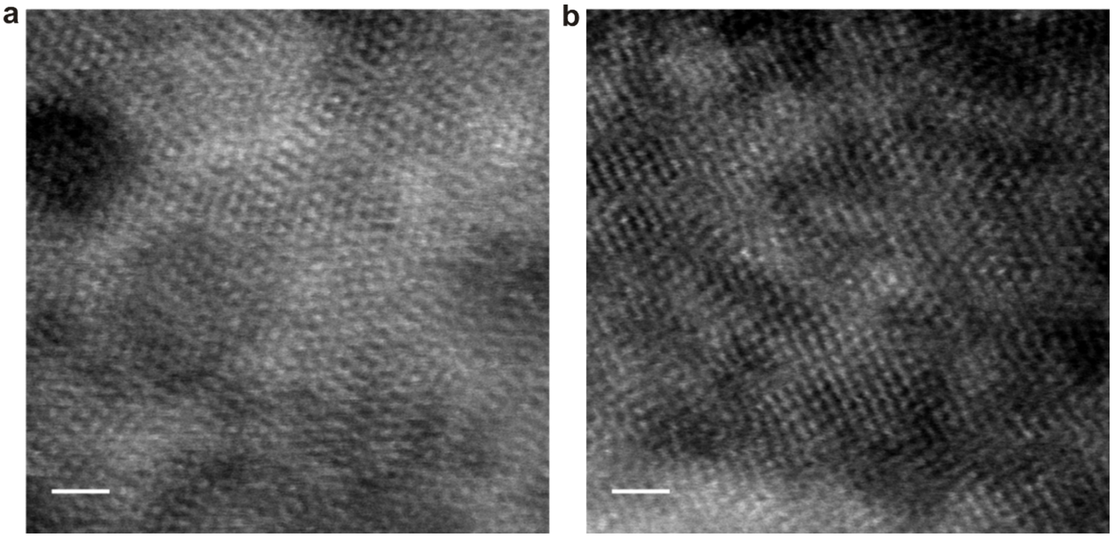

Supplementary Figure 9| Sub-angstron resolution aberration-corrected HAADF-STEM images. **a** The HAADF-STEM image of MoS_2. **b** The HAADF-STEM image of 1%Pd- MoS_2. The scale bars in the image indicate 1 nm in both figures.

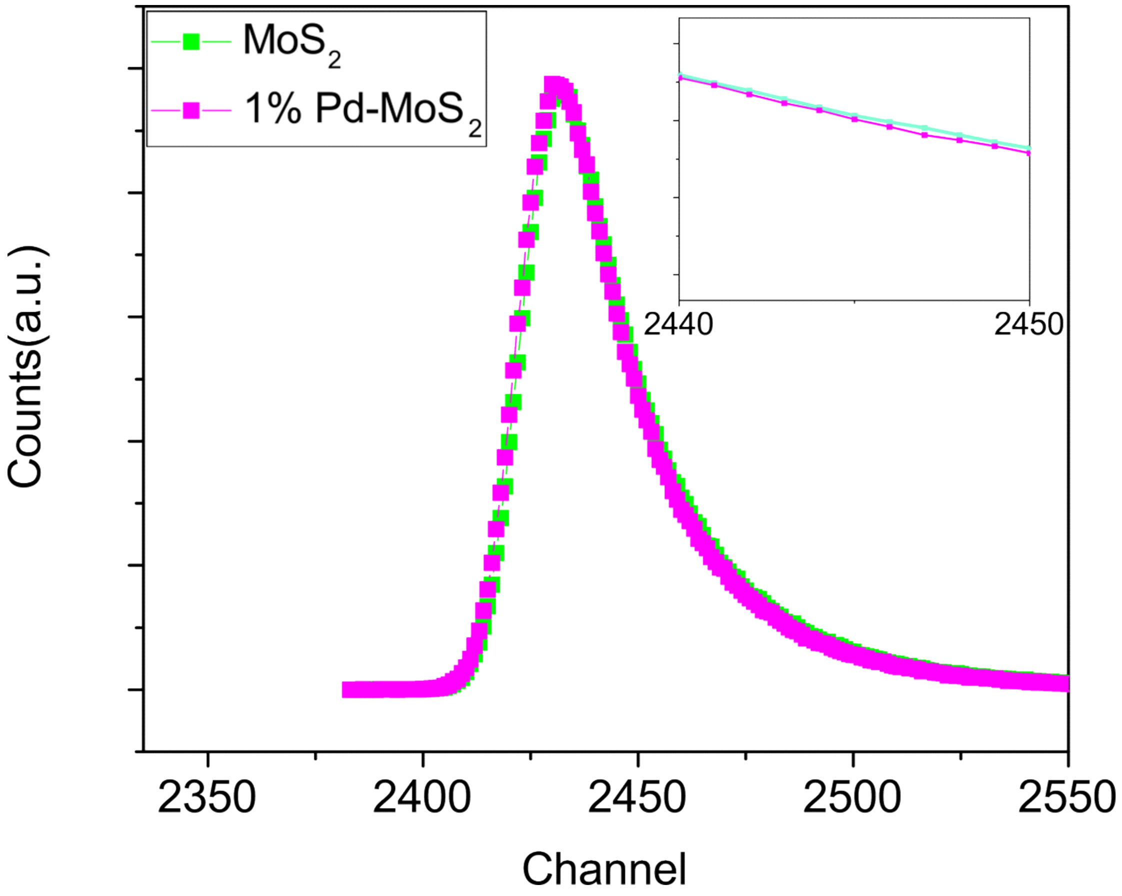

Supplementary Figure 10| PALS spectra. PALS spectra for 1%Pd-MoS_2 and MoS_2.

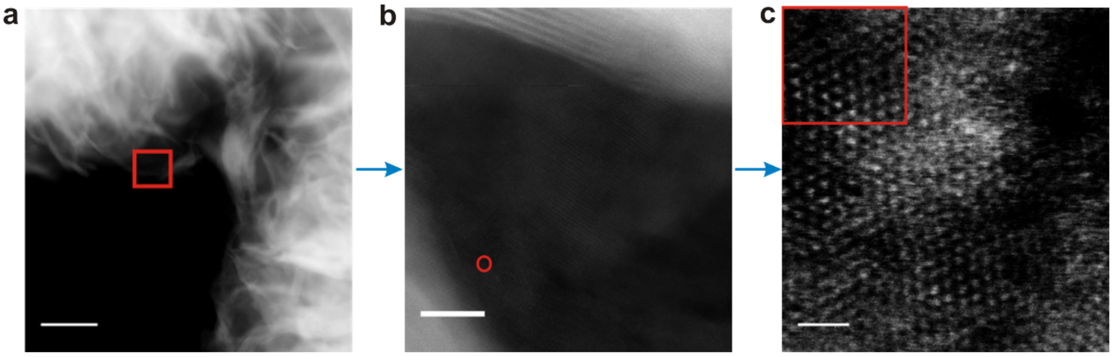

Supplementary Figure 11| STEM and HAADF-STEM images. a The STEM image of Pd-MoS_2. **b** HRTEM pattern corresponding mark. **c** The sub-angstron resolution aberration-corrected HAADF-STEM images of Pd-MoS_2 corresponding circle in **b,** here the marked tetragonal region represents the HAADF-STEM image of simple layer MoS_2. The scale bars in the images correspond to 100, 5, and 1 nm, respectively.

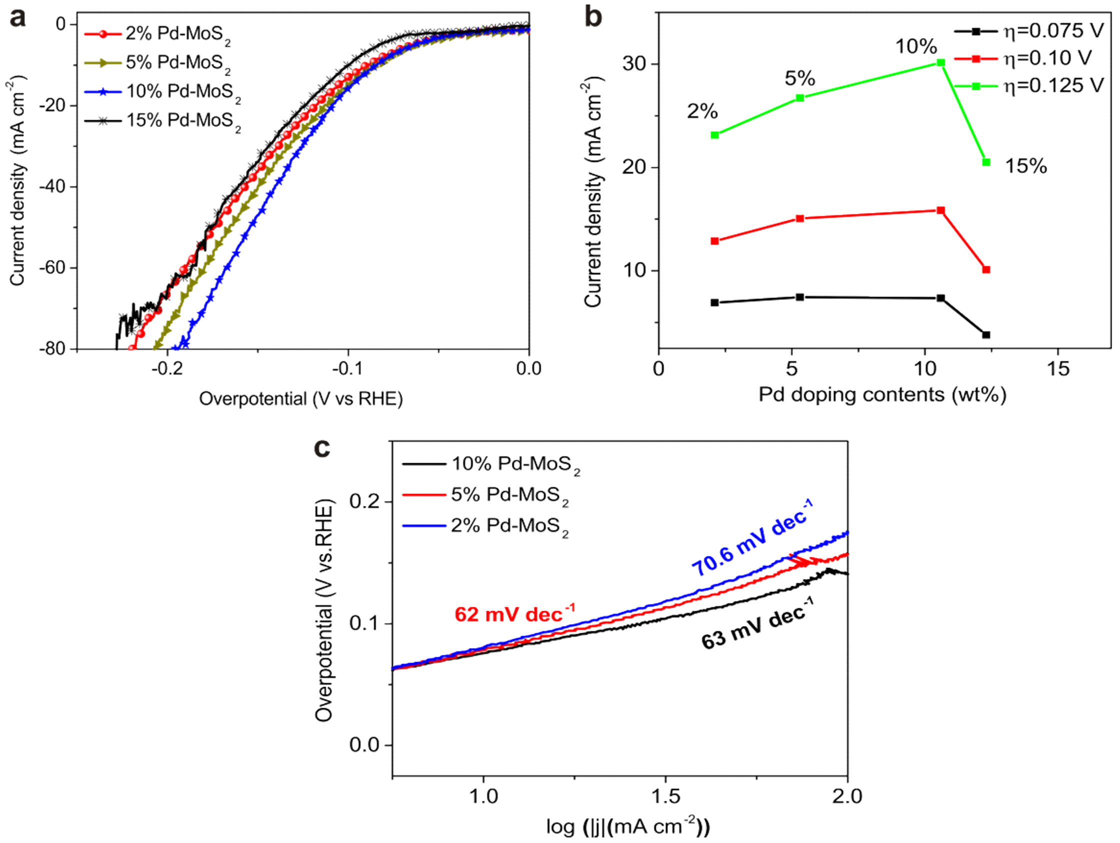

Supplementary Figure 12| Electrochemical activity of Pd-MoS$_2$ catalysts.

a Polarization curves of 2%Pd-MoS$_2$, 5%Pd-MoS$_2$, 10%Pd-MoS$_2$ and 15%Pd-MoS$_2$.

b Current densities at overpotential of 0.075 V, 0.1 V and 0.125 V for Pd-MoS$_2$ with different Pd doping contents. **c** Tafel plots derived from the results given in **a**.

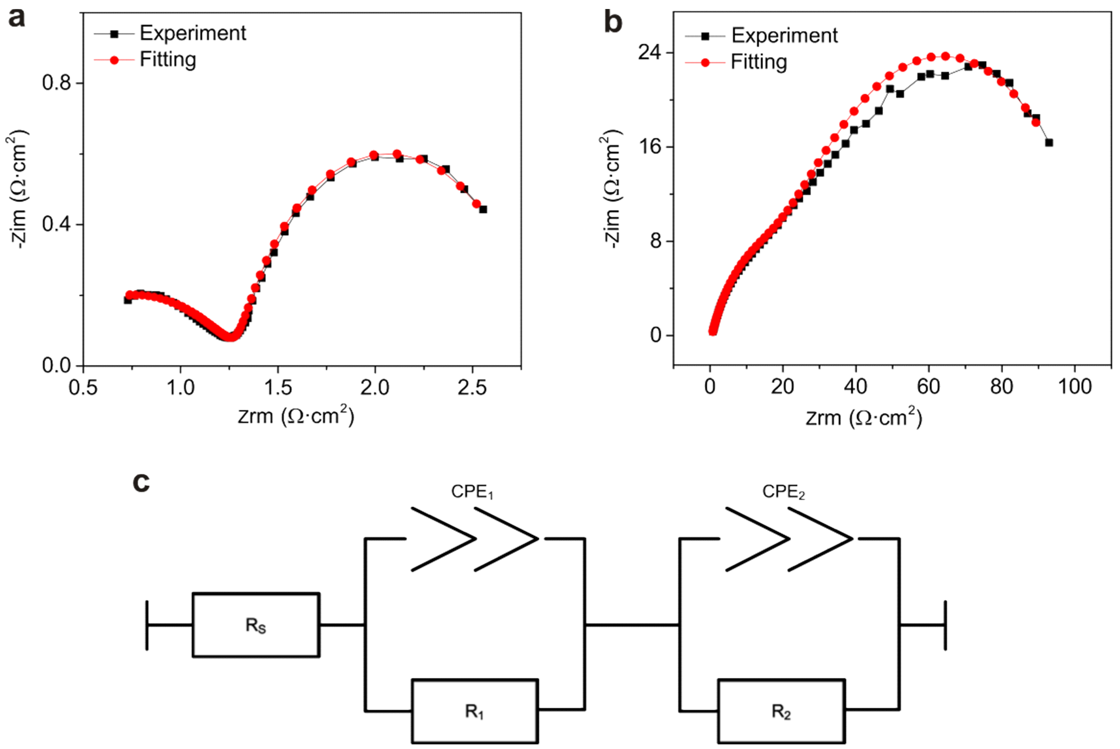

Supplementary Figure 13| EIS spectrum. a-b EIS comparison for 1%Pd-MoS_2 and MoS_2 in terms of Nyquist plots. The black solid lines are experimental results, and the red dotted lines are best-fit curves. **c** Equivalent circuit models used for fitting the EIS response of HER, where Rs is the electrolyte resistance, R_1 is related to the interface resistance, R_2 denotes the charge-transfer resistance, and CPE represents the constant phase element.

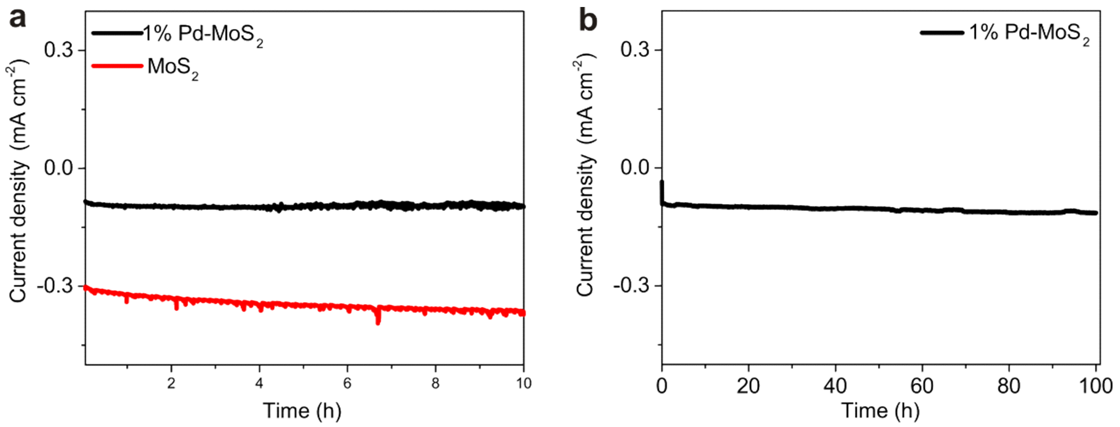

Supplementary Figure 14| Electrochemical stability. a Chronoamperometry (CP) tests of 1%Pd-MoS_2 and MoS_2 at a current density of 10 mA cm^{-2} (10 h). **b** CP tests of 1%Pd-MoS_2 at a current density of 10 mA cm^{-2} (100 h).

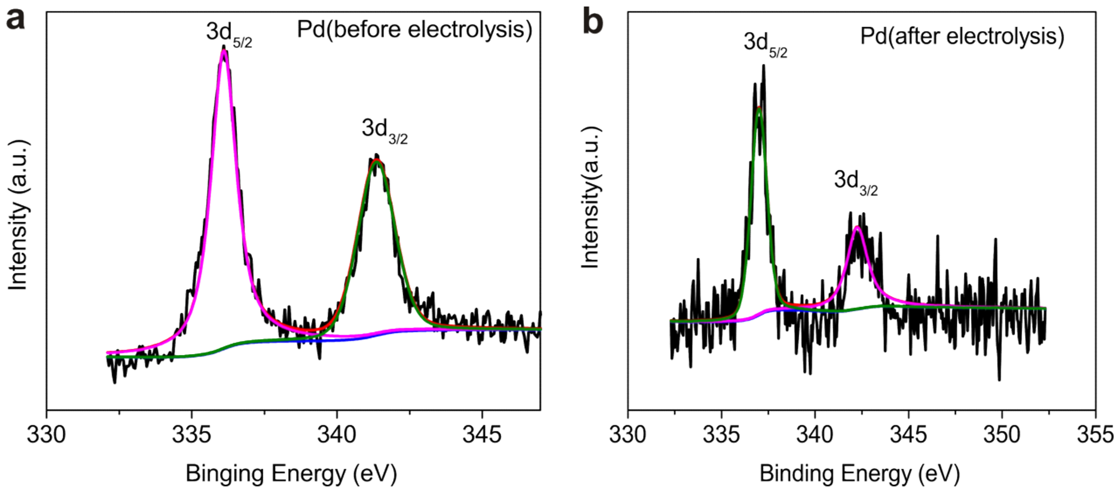

Supplementary Figure 15| XPS spectrum. a High-resolution XPS results (Pd 3*d* region) of the Pd-MoS_2 before electrolysis at 10 mA cm^{-2} for 100 h. **b** High-resolution XPS results (Pd 3*d* region) of the Pd-MoS_2 after electrolysis at 10 mA cm^{-2} for 100 h.

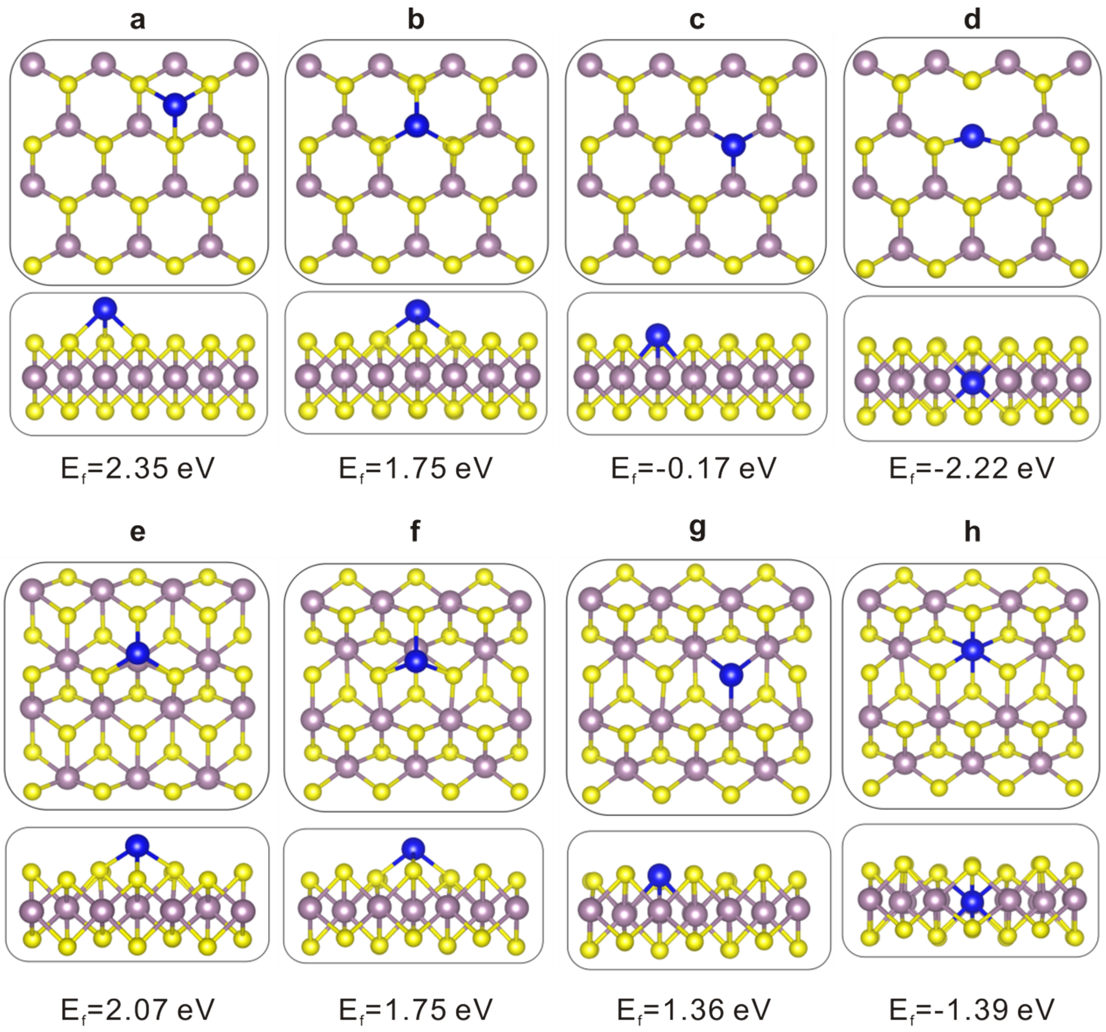

Supplementary Figure 16| Optimized atomic configurations of Pd atoms in 2H-MoS_2 and 1T-MoS_2. **a** The formation energy (E_f) of Pd atoms on hollow sites in 2H-MoS_2. **b** The formation energy (E_f) of Pd atoms on Mo atop sites in 2H-MoS_2. **c** The formation energy (E_f) of Pd atoms replace S sites in 2H-MoS_2. **d** The formation energy (E_f) of Pd atoms replace Mo sites in 2H-MoS_2. **e** The formation energy (E_f) of Pd atoms on hollow sites in 1T-MoS_2. **f** The formation energy (E_f) of Pd atoms on Mo atop sites in 1T-MoS_2. **g** The formation energy (E_f) of Pd replace S sites in 1T-MoS_2. **h** The formation energy (E_f) of Pd atoms replace Mo sites in 1T-MoS_2.

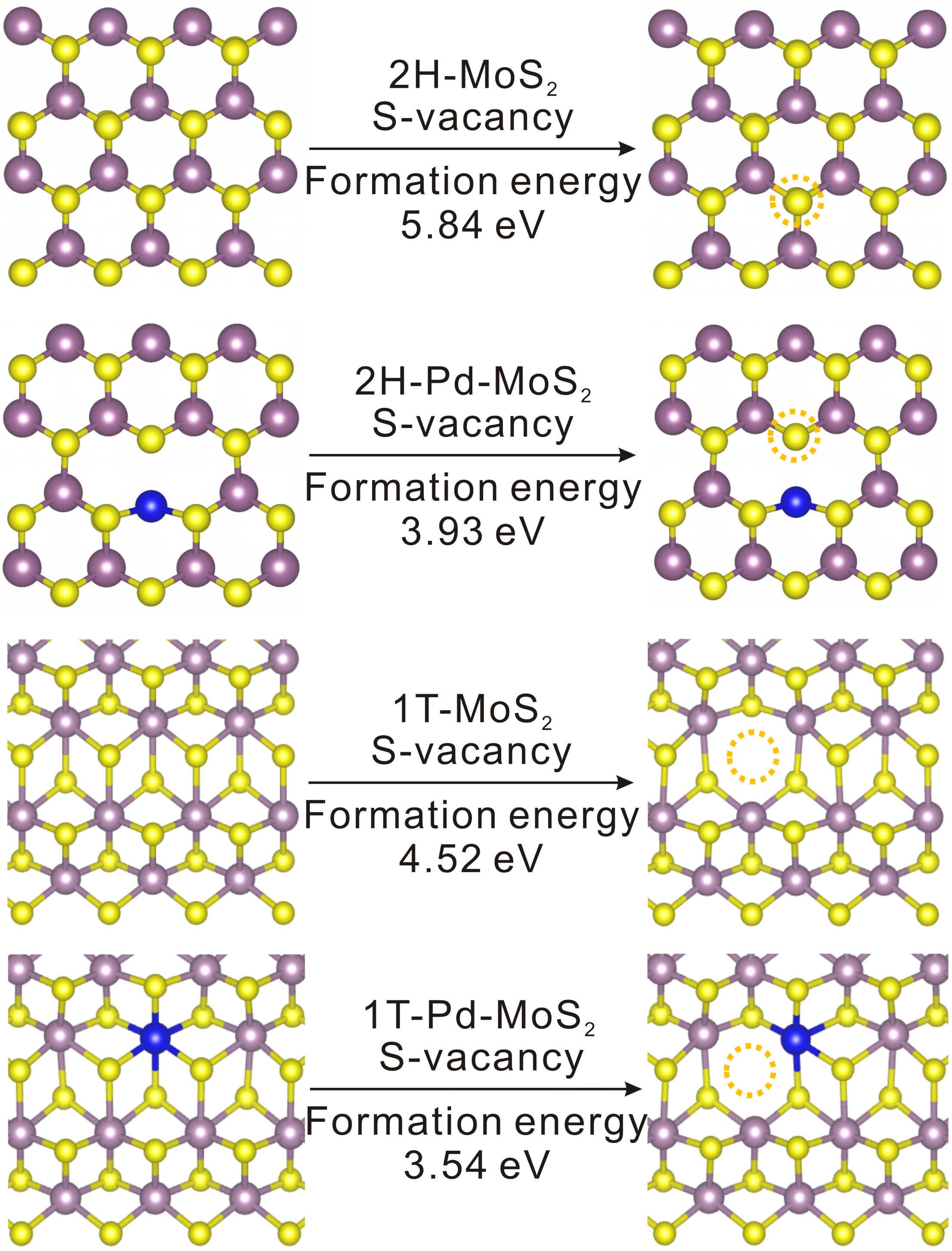

Supplementary Figure 17| Formation energy of S-vacancy. Formation of SVs in $2H\text{-}MoS_2$, $2H\text{-}Pd\text{-}MoS_2$, $1T\text{-}MoS_2$ and $1T\text{-}Pd\text{-}MoS_2$.

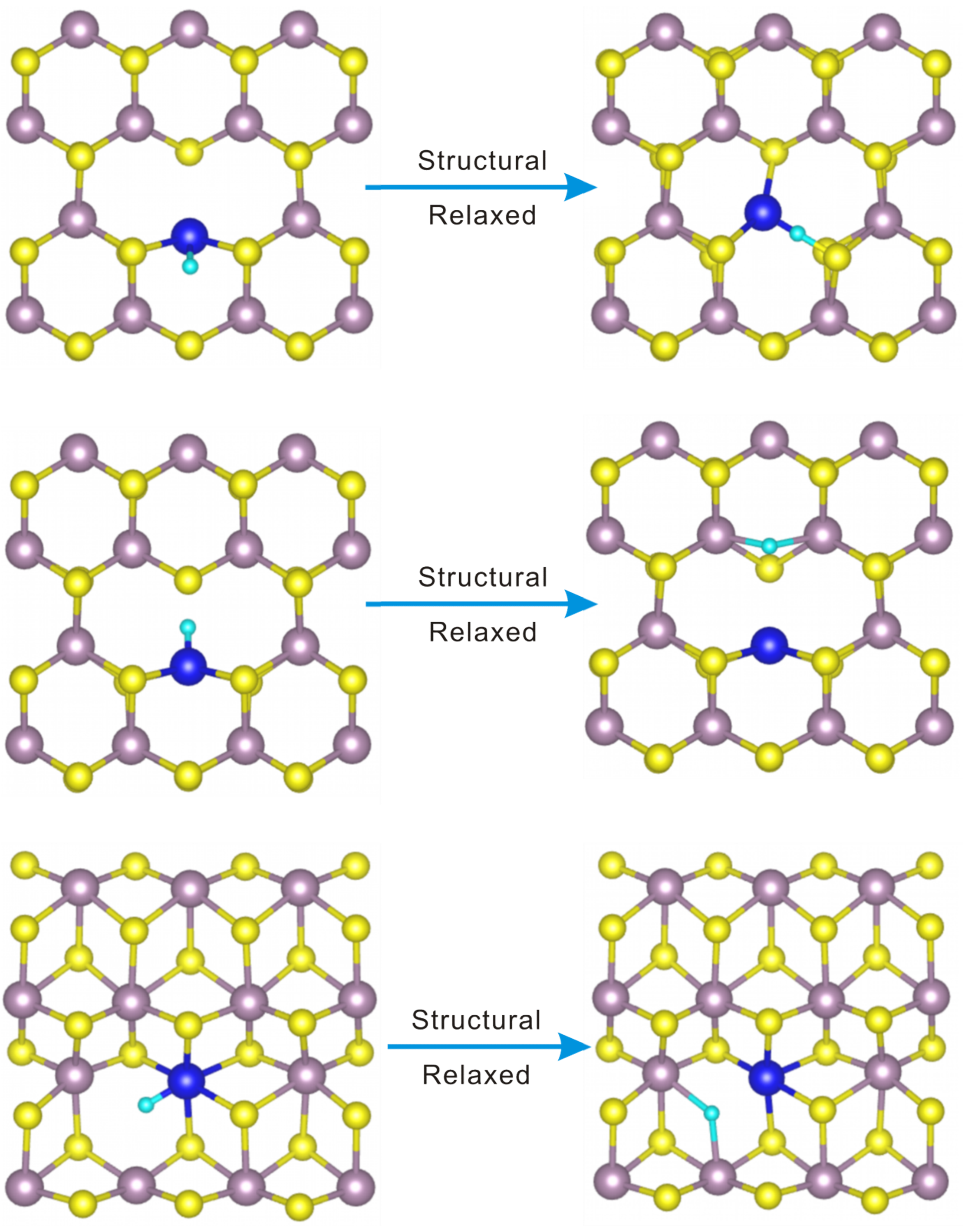

Supplementary Figure 18| H atom absorbing at Pd sites of 2H-Pd-MoS_2 and 1T-Pd-MoS_2.

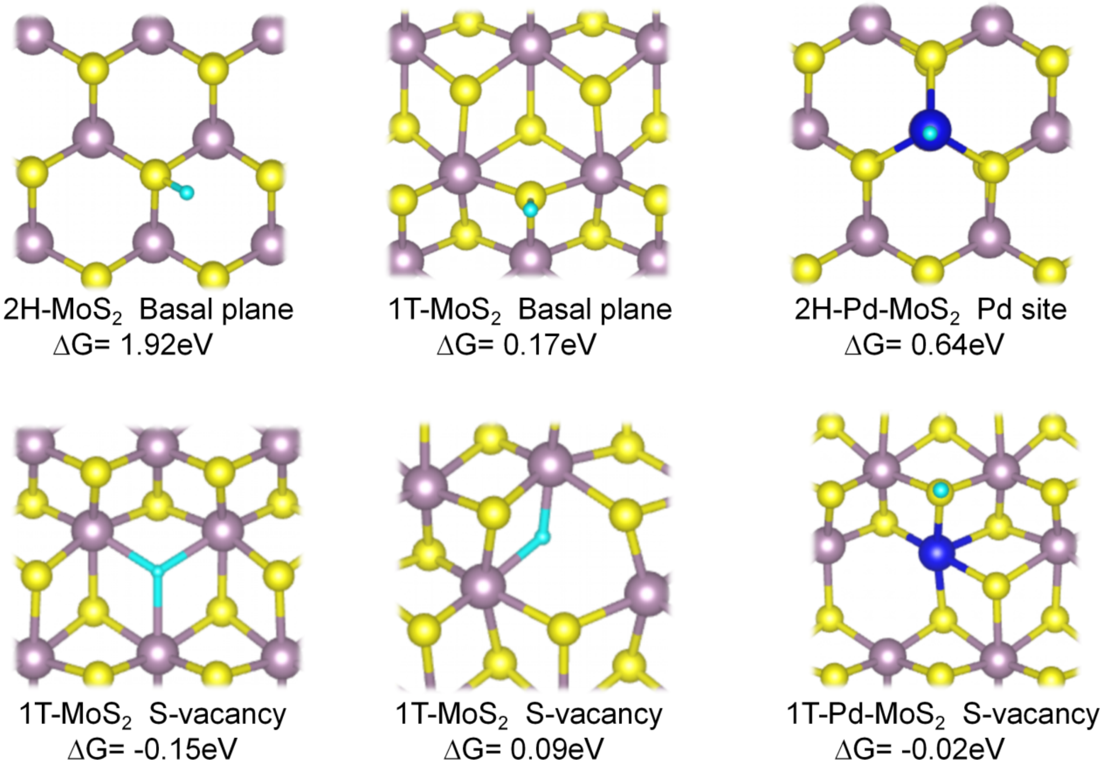

Supplementary Figure 19| H adsorption free energy. Adsorption positions for single H atom absorbing at basal plane of 2H-MoS_2, basal plane of 1T-MoS_2, Pd site of 2H-Pd-MoS_2, S-vacancy of 1T-MoS_2 and S atop site of 1T-Pd-MoS_2, and the adsorption free energies are given.

Supplementary Table 1. The element stoichiometry of MoS_2 and 1%Pd-MoS_2.

Crystalline	The stoichiometry of elements
MoS_2	1:1.87 (Mo:S)
1%Pd-MoS_2	1:1.82:0.02 (Mo:S:Pd)

Supplementary Table 2. The solubility product constants of the different sulfides.

Molecular formula	K_{sp}	Pk_{sp} (-lg k_{sp})
α-NiS	3.2×10^{-19}	18.5
β-NiS	1.0×10^{-24}	24.0
γ-NiS	2.0×10^{-26}	25.7
PdS	2.03×10^{-58}	57.69
FeS	6.3×10^{-18}	17.2
α-CoS	5×10^{-22}	21.3
β-CoS	3×10^{-26}	25.5

Supplementary Table 3. ICP-OES results of dissolved S, Mo, Pt, Au and Pd ions in solution after this redox process.

Dissolved ions into the solution (μg mL^{-1})	Mo	S	Pd	Au	Pt
Pd-MoS_2	70.02	41.18	0	-	-
MoS_2	0	0	-	-	-
Pt-MoS_2	82.17	0	-	-	0
Au-MoS_2	77.63	0	-	0	-

Supplementary Table 4. ICP-OES results of Pd in solution and samples in MoS_2 (2H) after reaction with Pd (Ⅱ)

The amount of Pd in solution or sample (μg mL^{-1} or wt %)	Pd
Solution before redox reaction	12.12
Solution after redox reaction	11.87
MoS_2 (2H) after reaction with Pd (Ⅱ)	Under detect limit

Supplementary Table 5. Pd L-edge EXAFS curves fitting parameters.

Sample	Shell	N	R(Å)	σ^2(10^{-3} Å^2)	ΔE_0(eV)	R-factor
Pd-MoS_2	Pd-S	4.3±0.3	2.33±0.01	3.9±0.7	1.3±0.8	0.006

N, coordination number; R, distance between absorber and backscatter atoms; σ^2, Debye-Waller factor; $\Delta E_{0,}$ inner potential correction accounting for the difference in the inner potential between the sample and the reference compound.

Supplementary Table 6. The fitting results about PALS.

Sample	τ_1(ns)	I_1(%)	τ_2(ns)	I_2(%)	τ_3(ns)	I_3(%)
MoS_2	0.1836	49.1	0.356	49.2	2.359	1.683
1%Pd-MoS_2	0.2062	57.5	0.385	40.8	2.309	1.736

Supplementary Table 7. Summary of peak area ratio, and degree of defects for 1%Pd-MoS_2 and MoS_2.

Sample	Peak area ratio (S:Mo)	Element (At %)			Degree of defects (%)
MoS_2	1.361	Mo: 17.98	S: 82.02		0
1%Pd-MoS_2	1.128	Mo: 20.2	S: 76.33	Pd: 3.47	16.7%

Supplementary Table 8. Comparison of HER activity for 1%Pd-MoS_2 with other MoS_2-based electrocatalysts in acidic media.

Catalyst	Electrolytes	η (mV vs RHE) for j = -10 mA cm^{-2}	Current density (μA cm^{-2})	References
1% Pd-MoS_2<br>1%Pd-MoS_2/CP	0.5 M H_2SO_4	89<br>78	805<br>-	This work
$CoMoS_3$-PHSMS	0.5 M H_2SO_4	171	11	*Adv.Mater.* **28**, 92-97 (2016)
MoS_2 flakes<br>MoS_2 films	0.5 M H_2SO_4	~250<br>~175	3.5<br>40	*J.Am.Chem.Soc.* **138,** 16632 (2016)
P-1T-MoS_2<br>P-2H-MoS_2	0.5 M H_2SO_4	153<br>218	15.8<br>10.5	*J.Am.Chem.Soc.* **138,** 7965 (2016)
P-2H-MoS_2+S<br>1T-MoS_2<br>2H-MoS_2		257<br>203<br>343	7.9<br>12.6<br>3.2	
V-MoS_2(EC)	0.5 M H_2SO_4	320	-	*Nat.Commun.* **8,** 15113 (2017)
MoS_2-BP	0.5 M H_2SO_4	85	-	*Nano lett.* **17,** 4311 (2017)
MoSSe	0.5 M H_2SO_4	300	-	*Nano Energy* **12,** 72211-2855 (2016)
Pt-MoS_2	0.1 M H_2SO_4	~140	-	*Energy. Environ. Sci.* **8**, 1594-1601 (2015)
SV-MoS_2<br>V-MoS_2	0.5 M H_2SO_4	170<br>250	-<br>-	*Nat. Mater.* **15,** 364 (2016)
Edge ox 1T MoS_2	0.5 M H_2SO_4	~200	-	*Nano lett.* **13**, 6222-7 (2013)
SL-MoS_2-CNTS	0.5 M H_2SO_4	~220	-	*RSC Advance* **4**, 34733 (2014)
M-MoS_2	0.5 M H_2SO_4	175	-	*Nat Commun.* **7**, 10672 (2016)
MoS_2/$CoSe_2$	0.5 M H_2SO_4	68	73	*Nat Commun.* **6**, 5982 (2015)

Supplementary Table 9. The EIS fitting results of 1%Pd-MoS_2 and MoS_2.

Sample	R_S (Ω cm^2)	R_1 (Ω cm^2)	R_2 (Ω cm^2)
MoS_2	0.51	18.38	92.89
1%Pd-MoS_2	0.18	1.14	7.5

Supplementary Table 10. The intrinsic activity of each site in 1%Pd-MoS_2.

	Total Current density (mA cm^{-2})	The Current density contributed by Pd doping (mA cm^{-2})	$\overline{TOF}$ (s^{-1})
0V	0.805	0.774	*0.15*
0.1V	14.756	14.756	2.77
0.15V	41.882	41.882	7.84
0.16V	48.622	48.622	9.10
0.2V	89.556	88.328	16.54
0.24V	114.33	112.96	21.15

Supplementary Table 11. The TOF of other MoS_2-based catalysts reported in the literature.

Catalyst	TOF (s^{-1}) at exchange current density	Overpotential (V)	Corresponding TOF (s^{-1})	Reference
MoS_2	0.013	-	-	*Nano lett.* **13**, 1341 (2013)
UHV-deposited MoS_2 nanoparticles on Au(III)	-	0.1	1	*Science* **317,** 100 (2007)
		0.16	10	
MoS_2 nanoparticles	0.02	-	-	
Sub-monolayer $[Mo_3S_{13}]^{2-}$ HOPG	-	0.2	3	*Nat.Chem.* **6**, 248-253 (2014) ,
$[Mo_3S_4]^{4+}$ cubanes/HOPG	0.07	0.3	1~5	*J.Phys.Chem.C.* **112**, 17492 (2018)
Amorphous MoS_3-CV film		0.22/0.24	0.8/2	*Chem.Sci.* **2**, 1262 (2011)
MoS_2	-	0.3	3.83	*J.Am.Chem.Soc.* **139**, 15479 (2017)
Zn-MoS_2	-	0.3	15.44	
Irradiated Au-MoS_2	-	0.3	8.76	*J.Am.Chem.Soc.* **137**, 7365 (2015)
Double-gyroid MoS_2	-	0.2	1	*Nat.Mater.* **11,** 963 (2012)

MoO_3-MoS_2 nanowires		0.272	4	*Nano lett.* **11,** 4168 (2011)
Irradiated Au-MoS_2	-	0.3	8.76	*J. Am. Chem. Soc.* **137,** 7365 (2015)
Amorphous MoS_3	-	0.2	0.3	*ACS Catal.* **2**, 1916 (2012)
Defect-Rich MoS_2 sheets	-	0.3	0.725	*Adv.Mater.* **25**, 5807 (2013)
V-MoS_2/Au	0.05~0.16	-	-	*Nat.Mater*. **15**, 364 (2014)

Supplementary Table 12. Comparison of HER activity for 1%Pd-MoS_2 with the 1T-MoS_2, the 2H-MoS_2 with S-vacancies and 1T-MoS_2 with S-vacancies in acidic media.

Catalyst	Electrolytes	η (mV vs RHE) for j = -10 mA cm^{-2}	Current density (μA cm^{-2})	References
1% Pd-MoS_2<br>1%Pd-MoS_2/ CP	0.5 M H_2SO_4	89<br>78	805<br>-	This work
P-1T-MoS_2<br>P-2H-MoS_2<br>1T-MoS_2	0.5 M H_2SO_4	153<br>218<br>203	15.8<br>10.5<br>12.6	*J. Am. Chem. Soc.* **138,** 7965-72 (2016)
2H-MoS_2 with S-vacancies	0.5 M H_2SO_4	320	-	*Nat. Commun*. **8,** 15113 (2017)
1T-MoS_2 with edges	0.5 M H_2SO_4	~200	-	*Nano lett.* **13**, 6222-7 (2013)
1T-MoS_2	0.5 M H_2SO_4	175	-	*Nat Commun*. **7**, 10672 (2016)
1T-MoS_2 with SVs	0.5 M H_2SO_4	200	-	*Sci. Rep*. **6**, 31092 (2016)
2H-MoS_2 with SVs	0.5 M H_2SO_4	170	40	*J. Am. Chem. Soc.* **138**, 16632 (2016)
1T-MoS_2	0.5 M H_2SO_4	~210	-	*J. Am. Chem. Soc.* **135**, 10274 (2016)

P-1T-MoS_2 refres to 1T-MoS_2 with abundant S-vacancies and edges (the degree of defects exceeds 83.7%); P-2H-MoS_2 refers to 2H-MoS_2 with abundant S-vacancies and edges.

Supplementary Note 1: To judge the spontaneity of the chemical reaction

(1)

$Mo^{4+} + e^- \rightarrow Mo^{3+}$ $\varphi^\theta Mo^{4+} / Mo^{3+} = -0.04$ V

$Pd^{2+} + 2e^- \rightarrow Pd$ $\varphi^\theta Pd^{2+} / Pd = 0.951$ V

$2Mo^{3+} + Pd^{2+} \rightarrow 2Mo^{4+} + Pd$

$E^\theta = \varphi^\theta Pd^{2+} / Pd - 2\varphi^\theta Mo^{4+} / Mo^{3+} = 0.951\ V – 2\times (-0.04\ V) = 1.031\ V$

$\Delta rG^\theta = -nE^\theta F = -2 \times 1.031 \times 96500\ J = -198.983\ kJ < 0$

(2)

$Pd^{2+} + 2e^- \rightarrow Pd$ $\varphi^\theta Pd^{2+} / Pd = 0.951$ V

$PdS \rightarrow Pd^{2+} + S^{2-}$ $ksp = 2.03\times10^{-58}$

$Pd + S^{2-} \rightarrow PdS + 2e^{-1}$ $\varphi^\theta PdS/Pd$

$$\varphi^\theta PdS / Pd = \varphi^\theta Pd^{2+} / Pd - \frac{RT}{nF}\ln\frac{1}{[Pd^{2+}]} = 0.951 - \frac{8.314\times 298}{2\times 96500}\times \ln\frac{1}{2.03\times 10^{-58}} = -0.754\ V$$

(3)

$PdS + 2e^- \rightarrow Pd + S^{2-}$ $\varphi^\theta PdS / Pd = -0.754$ V

$2Mo^{4+} + 2e^- \rightarrow 2Mo^{3+}$ $\varphi^\theta Mo^{4+} / Mo^{3+} = -0.04$ V

$Mo^{4+} + Pd + S^{2-} \rightarrow Mo^{3+} + PdS$

$E^\theta = -0.04V – (-0.754\ V) = 0.75\ V$

$\Delta rG^\theta = -nE^\theta F = -2\times0.75\times96500\ J = -144.75\ kJ < 0$

Supplementary Note 2: Verifying the reaction mechanism

We mixed the pristine MoS_2 sample (HM-MoS_2) with Pd (II), Pt (IV), and Au (III) complex solutions for reaction, respectively, and the oxidizability of Pt (IV) and Au (III) were intentionally tuned to be weaker than Pd (II) through complexation. On one hand, all of the Mo (III) was oxidized into Mo (IV), accompanied with the complete reduction of Pt (IV) and Au (III) into Pt (0) and Au (0) metallic states in the resultant Pt-MoS_2 and Au-MoS_2 hybrids. (Supplementary Fig. 1). Moreover, the leaching of Mo ions into the solution after redox reaction was measured through ICP-OES test (Supplementary Table 3), which well corresponding to what suggested from Equation ($4Mo^{3+} \rightarrow 3Mo^{4+} + 3e$(*left*) $+V_{Mo}^{///}$ (*one Mo left*)). Furthermore, the oxidation of all Mo (III) into Mo (IV) and the leaching of Mo ions (due to principle of conservation of charge) together unambiguously demonstrate that Mo in valence (III) can act as the reducing agent towards the reduction of Pt, Au, and Pd, etc. On the other hand, Pd is expectedly accommodated into MoS_2 sample as Pd (II), as confirmed by binding energies peaks at only 336.7 eV ($3d_{5/2}$) and 342 eV ($3d_{3/2}$) observed, using high-resolution X-ray photoelectron spectroscopy (Fig. 1b). The final Mo exhibits mixed valence states of III/IV, corresponding to the Mo (III) oxidation followed by Mo (IV) reduction, accompanied with the incorporation of Pd. The leaching of Mo ions and S ions into the solution was also detected by ICP-OES(Supplementary Table 3), further corresponding to redox reaction ($2Mo^{4+} + S^{2-} + 2e \rightarrow 2Mo^{3+} + V_S^{//}$ (one S left)). These results indicate the successful doping of Pd into MoS_2 backbone through spontaneous atomic Pd interfacial redox doping mechanism.

To further confirm our conclusion, we used the standard 2H MoS_2 sample (Mo: S=1:2, without Mo (III) species) instead of the chemically synthesized MoS_2 (homemade pristine MoS_2 with stoichiometry at 1:1.87) to react with Pd (II). As shown in Fig. 1a and Supplementary Fig. 2, the Mo L_3-edge XANES spectra and high-resolution XPS results (Mo 3*d* region) of 2H MoS_2 sample confirmed that all Mo atoms are present as Mo (Ⅳ). The reaction between standard 2H MoS_2 and Pd $(OAc)_2$ was controlled at the same condition as those illustrated in the Methods. The concentrations of Pd in the solution before and after the reaction were monitored through ICP-OES, as shown in Supplementary Table 4. Clearly, no obvious change in Pd concentration is observed. Meanwhile, the final 2H-MoS_2 sample after reaction with Pd was also tested through ICP-OES, where no Pd (under the detection limit) was obtained in the final sample. All these results confirm that standard 2H-MoS_2 is not capable of reducing Pd (Ⅱ), further validating that Pd atoms are introduced through the redox mechanism.

Supplementary Note 3: Aberration-corrected HAADF-STEM images of 1%Pd-MoS_2

Due to the fact that MoS_2 was chemically synthesized through a hydrothermal technique, the final sample shows a flower-like two-dimensional nanosheets structure with both monolayer and multilayer flakes being observed through high-resolution TEM (HRTEM) and atomic force microscopy (AFM). The multi-dispersion nature of our catalyst makes it showing different contrast in HAADF-STEM image. The brighter regions can be ascribed to the presence of multilayer MoS_2. The uniformly

darker regions can be found when the electron beam exposure time was over 60 s. In order to minimize such knock-on damage, the electron beam exposure time was limited to 20 s for each region of the specimen investigated. Furthermore, we have to mention that the Pd redox doping can induce more Mo vacancies and S vacancies, which results in regions where a perfect crystalline atomic lattice is not visible. (Supplementary Fig. 9 and Supplementary Fig. 11)

Supplementary Note 4: Pd doped configurations

Four possible doped sites of 2H-MoS_2 and 1T-MoS_2 were taken into account, as shown in Supplementary Fig. 16. In the case of 2H-MoS_2, a is Pd atoms above hollow sites, b is Pd atoms above Mo atom, c is Pd atom replacing S atom, and d is Pd atom replacing Mo atom. In case of 1T-MoS_2, e, f are Pd atom above Mo atom, g is Pd atom replacing S atom, and h is Pd atom replacing Mo atom. The formation energy (E_f) for these different structures were also calculated to evaluate their structural stability by $E_f = E_{tot} – E_{0[MoS2]} – E_{Pd}$,[1] where E_{tot} is the total energy for MoS_2 with doped or supported metal atoms. In the cases of metal doped MoS_2, $E_{0[MoS2]}$ is the energy for MoS_2 with one Mo or S vacancy, while for the cases of metal supported on MoS_2, $E_{0[MoS2]}$ is the energy for perfect MoS_2. E_{Pd} is the energy of one metal atom referred to its bulk metal. According to our calculations, when Pd atoms replace Mo atoms, the formation energy is the lowest.

Supplementary Note 5: Phase transition

Supplementary Fig. 17 shows that increasing of Pd-doping concentrations and S-vacancy concentrations both can promote phase transition of MoS_2. When the concentration of SVs is 1.67% and the concentrations of Pd atoms exceed 24%, 1T-MoS_2 becomes more stable than 2H-MoS_2. Moreover, when the concentration of SVs increase to 16.7% (close to the SV concentrations of experimental sample), the critical Pd-doping concentration of phase transition to reduce to 3%. According to previous reports, when the concentrations of SVs are around 15%, the catalytic activity of SVs is the highest[2]. However, the high concentrations of SVs will greatly reduce the electrical conductivity of MoS_2, and affect catalytic performance. Nevertheless, the increase content of of 1T metal phase causing by a small amount of Pd-doping well compensates the conductivity reduction of MoS_2, and makes the active sites get electrons more easily. In addition, it is well known that the in-plane surface of 1T-MoS_2 is catalytically active for the HER[3]. In literature, the traditional way to induce phase transitions is lithium insertion[4], and where the active sites of surface are covered by lithium and thereby results in activity loss. However, in our system, the surface of 1T-MoS_2 is fully exposed, with active sites of surface been fully utilized.

Supplementary Note 6: TOF calculation

The TOF was calcualted according the formula:

$$TOF = \frac{Total\ number\ H_2\ atoms\ per\ \sec ond}{Total\ number\ of\ active\ sites\ per\ unit\ area} = \frac{j/(2\times q)}{N}$$

Where q = 1.6×10^{-19} C is the elementary charge, and 2 accounts for 2 H atoms per H_2 molecule.

The DFT calculations suggest that the S atoms near the Pd dopants in the MoS_2 are the prior adsorption sites, but the sulfur vacancies also may be as catalytically active sites. In the following we use the number of the sulfur vacancies and the S atoms near the Pd dopants in the MoS_2 as the number of active sites, which can be estimated from the number of Pd.

The number of the sulfur vacancies is estimated using defect reaction.

$$N_{S-vacancy} = N_{Pd}$$

The 1%Pd-MoS_2 loading (on a glassy carbon electrode) was 222 μg cm^{-2}. The MoS_2 is highly dispersed as few-layer thick sheets. We assume in the following calculation that all Pd atoms are surface exposed.

From ICP the atomic percentages of Pd were determined to be 1%. Using the deposited mass, molar masses and above atomic percentages, the number of Pd atoms, the total S vacancies, and the TOFs were calculated to be:

$$N_{Pd} = \frac{222 \, {}^{ug}\!/_{cm^2}}{160.072 \, {}^{g}\!/_{mol}} \times 6.022\times10^{23} mol^{-1} \times 1\% = 8.35\times10^{15} cm^{-1}$$

$$N_{S-vacancy+Pd-S^*} = 1.67\times10^{16} \ \ cm^{-1}$$

TOF at 0 V: TOF= 0.15 s^{-1} (j=0.774 mA cm^{-2})

TOF at 0.1 V: TOF= 2.77 s^{-1} (j=14.756mA cm^{-2})

TOF at 0.15 V: TOF= 7.84 s^{-1} (j=41.882 mA cm^{-2})

TOF at 0.16 V: TOF= 9.10 s^{-1} (j=48.622 mA cm^{-2})

TOF at 0.2 V: TOF= 16.54 s^{-1} (j=88.328 mA cm^{-2})

TOF at 0.24 V: TOF= 21.15 s^{-1} (j=112.961 mA cm^{-2})

Supplementary Note 7: Generation of intrinsically more active S atop active sites (Pd-S* sites)

The average TOFs of 1%Pd-MoS_2 are 0.15 s^{-1}, 2.77 s^{-1}, 7.84 s^{-1}, 9.10 s^{-1}, 16.54 s^{-1} and 21.15 s^{-1} at 0 V, 0.1 V, 0.15 V, 0.16 V, 0.2 V and 0.24 V, respectively. (Supplementary Table 10). As far as we known, the highest $TOF_{S\text{-}vacancy}$ ever reported for MoS_2 was 0.05~0.16 s^{-1} at 0 V, where a well-defined monolayer MoS_2 was supported on Au substrate, thus making all the SVs in the catalysts available at the reaction interface.[2] For all other catalysts represented multicrystalline or amorphous structures, much inferior catalytic behaviors were observed. For our 1%Pd-MoS_2 catalyst, it not only shows the highest TOF among all the multicrystalline or amorphous structured catalysts, but also represents TOF of 0.15 s^{-1} at 0 V, on the high end of the values reported in literature. (see Supplementary Table 11 for details) Taking into the account of the multicrystalline nature of our testing electrode, we can confirm that the much higher average TOF was obtained. Thus, the high average TOF of the 1%Pd-MoS_2 reflect that the increased catalytic activity is originated from both increase in active site density and the intrinsic activity of each site.

In addition, we also adopted another strategy by comparing the activity of our catalysts with the highest value reported for the 1T-MoS_2, the 2H-MoS_2 with S-vacancies and 1T-MoS_2 with S-vacancies. (1) The lowest overpotential reported for

$1T-MoS_2$ at 10 mA cm^{-2} is ~200 mV; (2) As far as we known, the best activity of $2H-MoS_2$ with S-vacancies is reported by Linyou Cao, et al[5],where the catalysts with optimal S-vacancies exhibits overpotential of 170 mV at 10 mA cm^{-2}. The catalyst shows a maximum catalytic activity with SV densities vary between 7%~15%, and further increasing the SV concentration leads to performance decay.[6] (3) Even in the case of $1T-MoS_2$ with abundant S-vacancies and edges (the degree of defects exceeds 83.7%), the lowest overpotential ever reported is 153 mV[5]. In our work, the $1\%Pd-MoS_2/CP$ exhibit very low overpotential at 10 mA cm^{-2} of only 78 mV, which is 75 mV smaller than the most active MoS_2 catalysts reported shown above. With the Tafel slope measured at 80 mV dec^{-1}, the decrease in overpotential corresponds to approximately 10 times increase in actives site density, provided that the sites only exhibits the same intrinsic activity with that of the S-vacancies and edges in $1T-MoS_2$. This is impossible as the degree of the defects in the reported sample is already very high (83.7%) and the catalyst loading used for tests were similar between our tests (0.15 mg cm^{-2} vs 0.22 mg cm^{-2}). (see Supplementary Table 12 for details) Therefore, it is actually the huge improvement in catalytic activity that leads us to look for the active sites with high intrinsic activity, where DFT calculations were adopted and the Pd adjacent S atoms were confirmed as supreme active sites towards HER.

Furthermore，the effect of Pd doping can also be clearly evidenced by comparing the catalytic activity before and after Pd doping. It is noted that electron paramagnetic resonance (EPR) can be used to measure unpaired electrons on coordinatively unsaturated defective sites and reflect basal sulfur vacancies present in these layers[7].

We have also compared the EPR signals before and after Pd doping. As shown in Fig. 2f, the 1%Pd-MoS_2 exhibits approximately 3 times as many unsaturated sites than the pristine MoS_2. However, the overpotential (20 mA cm^{-2}) was reduced by 266 mV, corresponding to an increase in active site density by 2113 times (calculated from the Tafel slope), if the increase in site density is the only reason for the catalytic performance enhancement. This huge difference unambiguously points to the generation of more active HER sites after Pd doping.

Supplementary Methods

Electrical resistances measurements. The electrical resistances of samples were determined by using a homemade button cell. The powder samples were pressed in the mold with certain pressure and time (~10 MPa, 5 min). The sample is inserted between two smooth polished steel discs. Electrochemical impedance spectroscopy (EIS) performed at high frequency using Princeton Applied Research PARATAT MC. The operating frequency range was between 10 mHz and 10 kHz, the DC potential was 0 V compared to an open circuit, and the AC amplitude was 10 mV. In this case, the phase angle between the voltage applied and the current induced is zero; the impedance of the sample as a function of frequency is present as a horizontal line. The value of resistance of sample is equal to the impedance; and the resistance can be directly read from the |Z| -axis in the Bode. The electronic conductivity was calculated according the formula:

$$\sigma = \frac{l}{RS}$$

Here, the l is the thickness of specimen, R is electrical resistance of specimen, S is the area of specimen. l and S can get from the mould. R is obtained from Bode spectra (Bode spectra are read from the electrochemical impedance spectroscopy).

Computational details. The formation energy (E_f) for different S-vacancies were calculated to evaluate their structural stability, according to the formula $E_f = E_{SV} + E_S - E_{MoS2}$, where E_{SV} is the total energy for MoS_2 with S-vacancy, E_s is the energy for single S atom and E_{MoS2} is the total energy for MoS_2 without vacancy, respectively. The Gibbs free energy of H adsorption is calculated by $\Delta G_H = \Delta E_H + T\Delta S + \Delta E_{ZPE}$. ΔE_H is the adsorption energy of the H atom. $T\Delta S$ is the gas-phase entropy contribution of a hydrogen molecule at 298 K (It is a constant, 0.40 eV). ΔE_{ZPE} is the zero-point energy difference between the adsorbed state of the system and the gas phase state. "Additionally, the potential dependence of hydrogen adsorption is including using the computational hydrogen electrode model[8, 9] , where $\Delta G_{H^+}(aq) + \Delta G_{e-} = 1/2\Delta G_{H(aq)}$ at a potential of U=0 V versus RHE.

Supplementary References

1. Deng, J. et al. Triggering the electrocatalytic hydrogen evolution activity of the inert two-dimensional MoS_2 surface via single-atom metal doping. *Energy Environ. Sci.* **8**, 1594-1601 (2015).
2. Li, H. et al. Corrigendum: Activating and optimizing MoS_2 basal planes for hydrogen evolution through the formation of strained sulphur vacancies. *Nat.*

Mater. **15**, 364 (2016).

3. Tang, Q. & Jiang, D.E. Mechanism of Hydrogen Evolution Reaction on 1T-MoS_2 from First Principles. *Acs Catal.* **6**, 4953-4961 (2016).
4. Chou, S.S. et al. Controlling the metal to semiconductor transition of MoS_2 and WS_2 in solution. *J. Am. Chem. Soc.* **137**, 1742-1745 (2015).
5. Yin, Y. et al. Contributions of phase, sulfur vacancies, and edges to the hydrogen evolution reaction catalytic activity of porous molybdenumdisulfide nanosheets. *J. Am. Chem. Soc.* **138**, 7965-7972 (2016).
6. Li, G. et al. All The Catalytic Active Sites of MoS_2 for Hydrogen Evolution. *J. Am. Chem. Soc.* **138**, 16632-16638 (2016).
7. Liu, G. et al. MoS_2 monolayer catalyst doped with isolated Co atoms for the hydrodeoxygenation reaction. *Nat. Chem.* **9**, 810-816 (2017).
8. Norskov, J.K. et al. Origin of the overpotential for oxygen reduction at a fuel-cell cathode. *J. Phys. Chem. B* **108**, 17886-17892 (2004).
9. Peterson, A.A., Abild-Pedersen, F., Studt, F., Rossmeisl, J. & Norskov, J.K. How copper catalyzes the electroreduction of carbon dioxide into hydrocarbon fuels. *Energy Environ. Sci.***3**, 1311-1315 (2010).
